# Regularised Finite-Element Model Updating of a Thin-Walled Cylinder Based on Non-Contact Experimental Modal Analysis

**DOI:** 10.3390/s26216755

**Published:** 2026-10-25

**Authors:** Miaoshuo Li, Yiming Ma, Yupeng Xia, Kaibo Lu, Chan Xu, Fengshou Gu, Qiuxia Fan

**Affiliations:** 1 School of Automation and Software Engineering, Shanxi University, Taiyuan 030031, Chinamayiming2@sxu.edu.cn (Y.M.);; 2 Precision and Vibration Control Innovation Team, Shanxi University, Taiyuan 030031, China; 3 Quantum Precision Measurement and Vibration Control Innovation Center, Taiyuan 030031, China; 4 School of Mechanical Engineering, Taiyuan University of Technology, Taiyuan 030024, China; 5 School of Computing and Engineering, University of Huddersfield, Huddersfield HD1 3DH, UK; f.gu@hud.ac.uk

**Keywords:** finite-element model updating, vision-based vibration measurement, thin-walled cylinder, Tikhonov regularisation, parameter degeneracy

## Abstract

Material removal changes the dynamics of thin-walled parts during machining, making an accurate pre-machining baseline essential for subsequent prediction. This study integrates non-contact testing, scan-based reconstruction and material-property updating into a free-state modelling workflow: laser vibrometry identifies the target frequencies, vision-based measurement extracts the end-face circumferential shapes, and three-dimensional scan data are reconstructed into editable CAD geometry. With geometry and Poisson’s ratio fixed, Young’s modulus *E* and density ρ are updated using a mean-square relative-frequency-error objective with Tikhonov regularisation, the weight being chosen by a discrete L-curve. Free-state frequencies identify the ratio E/ρ; regularisation selects a representative material-parameter pair under the specified prior and weight, while mode shapes provide morphological checks. In the case study on a thin-walled steel cylinder, the reconstructed model has a median absolute radial deviation of 0.0362 mm (about 1% of the mean wall thickness); the relative RMSE of the three target modes decreases from 1.71% to 1.00%, and that of the non-target modes excluded from the fit decreases from 0.68% to 0.65%, indicating comparable sub-percent aggregate errors with a small decrease for the examined free-state non-target modes. At a fixed weight, runs from two opposite initial directions converge to nearly identical material parameters, showing similar endpoints for the two examined runs under their respective search settings. Geometric, measurement and fitting errors are distinguished. Experimental assessment addresses the free state, complemented by a numerical demonstration under prescribed clamping conditions.

## 1. Introduction

Thin-walled cylindrical parts are lightweight and compact, and are widely used in aerospace, marine and petrochemical industries [1]. Their small wall thickness, low radial stiffness and weak damping make them prone to vibration and machining chatter, which degrade machining accuracy and surface quality; chatter prediction and suppression have therefore long been central topics in thin-walled manufacturing [2]. Moreover, material removal continuously alters the geometry, mass and stiffness of the workpiece, so its modal parameters vary throughout machining; this time-varying behaviour complicates chatter prediction and places higher demands on model accuracy [3].

The vibration of thin-walled cylindrical shells has been studied extensively: shell vibration theory and experiments were systematically summarised by Leissa [4], homogeneous shell dynamics were reviewed by Qatu [5], and fundamental-frequency approximations, classical theories and finite-element results were compared for thin cylinders [6]. Axial thickness variation, non-circular cross-sections and geometric imperfections all alter the natural frequencies and mode shapes [7,8,9], and the accuracy of modal parameters directly governs the reliability of chatter prediction [3].

Theory and simulation describe shell modal behaviour using geometric, material and boundary-condition inputs that carry measurement and modelling uncertainties. Using experimental data to identify and update these parameters has therefore become an important route to accurate modal prediction [10]. Its reliability rests on two factors: accurate experimental modal measurement and reliable finite-element modelling and updating algorithms.

Accelerometers with impact hammers or shakers are established tools for experimental modal analysis [11]. However, their added mass can alter the dynamics of lightweight thin-walled structures, and sparse measurement points limit spatial resolution. Laser Doppler vibrometry (LDV) provides accurate non-contact measurements [12], although point-by-point scanning limits full-field efficiency and increases cost. Vision-based methods use pixel arrays to resolve spatial vibration without added mass [13]. Recent motion-magnification studies describe advances in video-based vibration measurement [14,15]. Phase projection and wavelet denoising improve the identification of weak responses [16]. A review of vision-based displacement measurement discusses the effects of illumination, image noise and observation conditions [17]. This study therefore combines spatially resolved vision measurements with LDV frequency measurements to identify the target modes.

Finite-element model updating must consider geometric, material and boundary-condition errors together. Geometric deviations may be absorbed into material estimates, making measured geometry an important input. Laser scanning, structured light and stereo vision acquire surface point clouds [18], which reverse engineering converts into editable geometry [19]. The recent Point2CAD method combines point-cloud segmentation, surface fitting and topology recovery to reconstruct structured CAD models [20]. Recent work also addresses the generation of FE models from scanned data and reverse engineering for digital reconstruction [21,22]. For thin-walled cylinders, preserving the inner and outer contours and spatial thickness requires specific treatment. Here, layered sections and separate contour lofting are used, and radial CAD-STL deviations assess reconstruction consistency.

Existing reviews address parameter selection, objective functions and solution methods for model updating [23]. Sensitivity methods based on frequency and mode-shape residuals are widely used [24,25,26]. Bayesian methods describe parameter and model uncertainties [27]; a recent review also examines measurement errors, model-class selection and uncertainty quantification [28]. Hierarchical Bayesian updating further addresses modal-residual weighting [29]. Parameter identifiability depends on the information supplied by the available observations [30]. Regularisation complements the observation-based constraints with prior information to stabilise the inverse problem [31,32]. Recent work examines regularisation in structural model updating with noisy data [33]. With geometry, Poisson’s ratio and free boundaries fixed, natural frequencies identify E/ρ; the separate identification of Young’s modulus and density benefits from independent material information.

The objective of this study is to establish and experimentally assess a pre-machining free-state baseline model of the thin-walled cylinder through frequency prediction and end-face mode-shape agreement. Experimental assessment addresses the free state, and a supplementary calculation demonstrates the response to prescribed clamping conditions. Material-removal and cutting-response predictions form subsequent extensions. The assessed geometric and dynamic baseline provides a starting model for these extensions. First, LDV under flexible supports identifies frequencies, while high-speed vision determines end-face shapes and circumferential orders. Second, scanned inner and outer contours and spatial thickness are reconstructed into a solid FE model, with reconstruction and mesh effects assessed separately. Third, three target frequencies support Tikhonov-regularised material updating and analysis of E/ρ degeneracy. Two runs from opposite initial directions are compared. Non-target frequencies and end-face shapes provide checks within the free state; subsequent work will extend predictive validation to clamped and post-removal states.

## 2. Non-Contact Experimental Modal Analysis

To avoid the added mass of contact sensors on the thin-walled structure, vibration is measured in this study with a high-speed camera and laser Doppler vibrometry (LDV). The LDV records the radial response at a single wall point, while the camera records the end-face vibration from which end-face mode shapes and frequencies are extracted; the two are combined for target-mode matching.

### 2.1. Test Setup and Realisation of the Free Boundary

**Figure 1:**
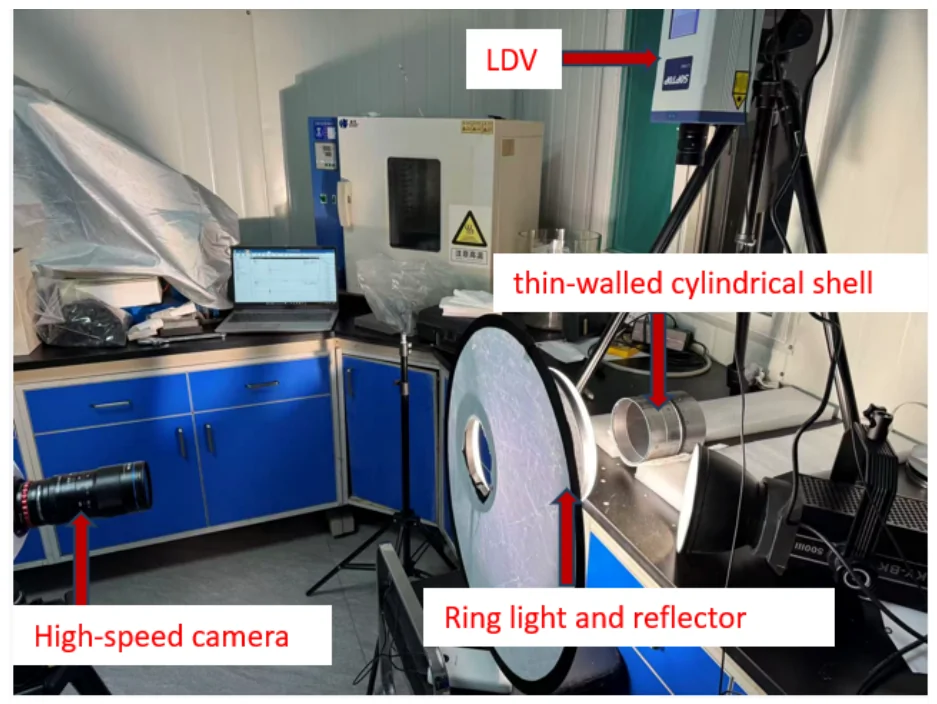
Non-contact modal measurement setup of the thin-walled cylinder.

The specimen is a thin-walled steel cylinder supported on flexible sponge pads to approximate free–free boundaries and reduce the influence of rigid supports on the modal test. The test system is shown in Figure 1: the specimen is excited by an impact hammer, and a high-speed camera (Neo 25M/C, Qianyanlang, Hefei, China) facing the end face records 1024×1024-pixel images at 6000 frames/s with a 47 µs exposure. The corresponding Nyquist frequency of 3000 Hz covers the highest target mode (2556.9 Hz). This sampling setting satisfies the sampling requirement for the target band; a ring light and reflector enhance the grayscale contrast of the end-face edge. A laser Doppler vibrometer (LV-P600HS, Shunchuang, Yuyao, China) records the single-point response simultaneously under the same state and excitation.

### 2.2. Full-Field Vibration Measurement by Vision

**Figure 2:**
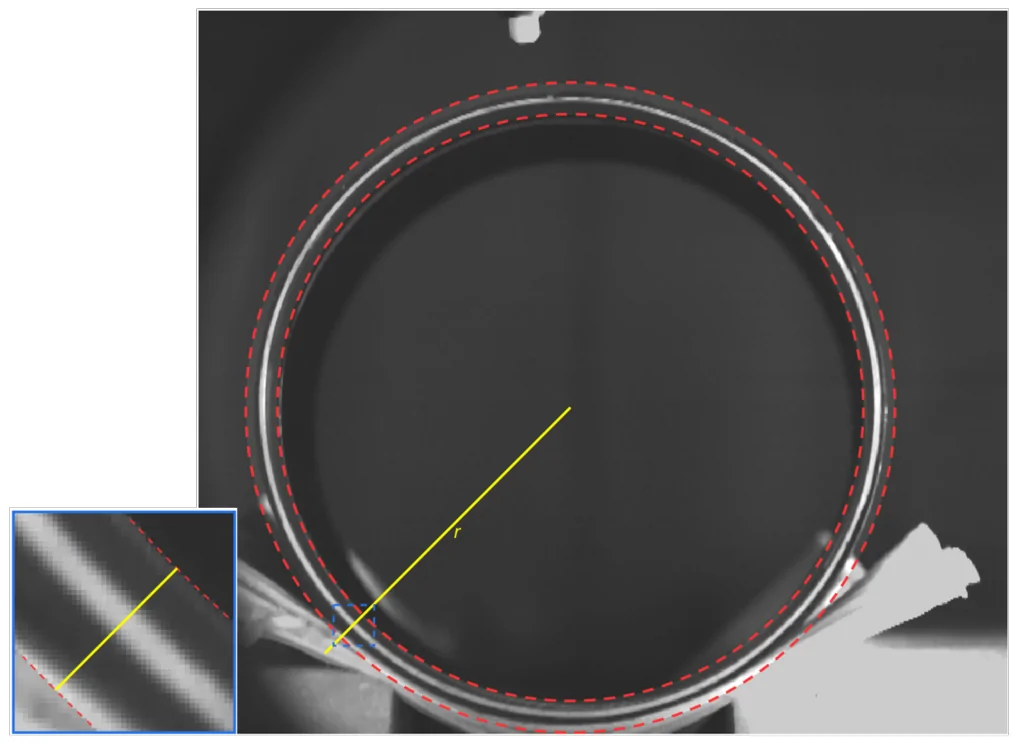
End-face sensing region and radial grayscale gradient for high-speed vision measurement. Red dashed circles bound the annular sensing region; the yellow line indicates the radial direction, and the blue box marks the magnified area.

The camera faces the annular edge of the end face, where pre-drawn black-and-white stripes form a radial grayscale edge distributed along the circumference (Figure 2, red dashed region). Following the pixel-array framework [34], pixels with large radial grayscale gradients on the inner and outer edges form the sensing array, organised by circumferential angle with radially outward taken as positive displacement.

**Figure 3:**
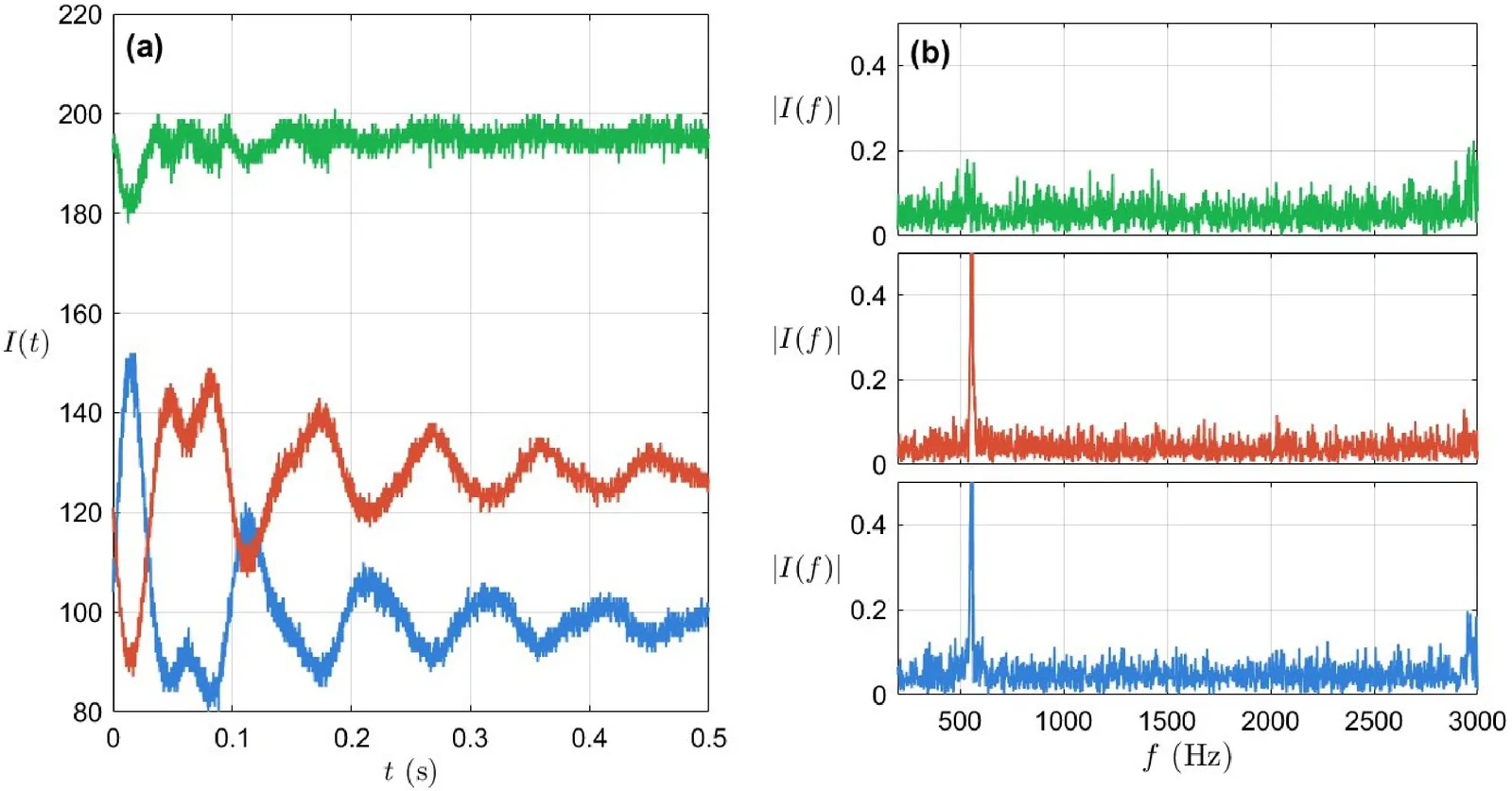
Vibration responses of representative pixels at different radial positions: (**a**) grayscale time histories I(t); (**b**) one-sided amplitude spectra |I(f)|. The horizontal axes are time (s) and frequency (Hz), respectively. The blue, green and red curves denote the responses of the inner-edge, middle and outer-edge pixels, respectively.

For small displacement and approximately constant local brightness, the response is extracted from the linear relation between grayscale variation and radial displacement. Figure 3 compares the responses of representative pixels at the inner edge, mid-radius and outer edge: (a) grayscale time histories I(t); (b) one-sided amplitude spectra |I(f)|. The edge pixels show pronounced responses and spectral peaks, whereas the mid-radius pixels are weaker; selecting high-gradient edge pixels therefore improves the sensitivity to radial vibration.

Following grayscale-response extraction, optimal phase-projection wavelet denoising (OPWD) [16] recovers the spatial end-face shape at each candidate frequency through the following steps.

1. The radial response time histories of the valid pixels are Fourier transformed to obtain the complex spectral coefficient $c_{j}\left(f\right)=a_{j}\left(f\right)+ib_{j}\left(f\right)$ at the candidate frequency *f*. Here *j* indexes the circumferential measurement points, and $a_{j}\left(f\right)$ and $b_{j}\left(f\right)$ are the real and imaginary parts of the coefficient, respectively. The coefficients are arranged in circumferential order to form the complex spatial response vector c(f).
2. To determine the unknown overall phase, projection angles are searched at $1^{\circ }$ steps over α∈[0,π). For each angle, uα=Re[c(f)e−iα] is denoised using step (4). The angle giving the highest mean power of the denoised spatial response is selected as $\alpha^{*}$.
3. The signed spatial response $u_{\alpha^{*}}$ corresponding to the optimum angle is selected. Angles differing by π yield equivalent shapes with opposite overall signs, preserving relative signs between measurement points and MAC. A fixed reference point standardises the overall sign for display. Phase projection recovers the spatial response, while subsequent circumferential rotational registration aligns the spatial orientations of the experimental and FE shapes.
4. The projected circumferential sequence is periodically extended to join its two ends and is then decomposed into five levels using the sym6 wavelet. The false discovery rate (FDR) criterion adaptively determines the hard threshold for the detail coefficients at each level, with a common FDR control level of Q=0.01. The noise standard deviation is estimated from the median absolute finest-level detail coefficient divided by 0.67449. Centred and normalised coefficients at each level yield two-sided Gaussian *p*-values, which are sorted in ascending order. At level *ℓ*, the largest index satisfying $p_{\left(j\right)}\leq Qj/M_{\ell }$ determines the hard threshold, where $M_{\ell }$ is the number of detail coefficients at that level. Detail coefficients below the threshold are set to zero, and the coarsest approximation is retained for reconstruction of the denoised spatial response.

**Figure 4:**
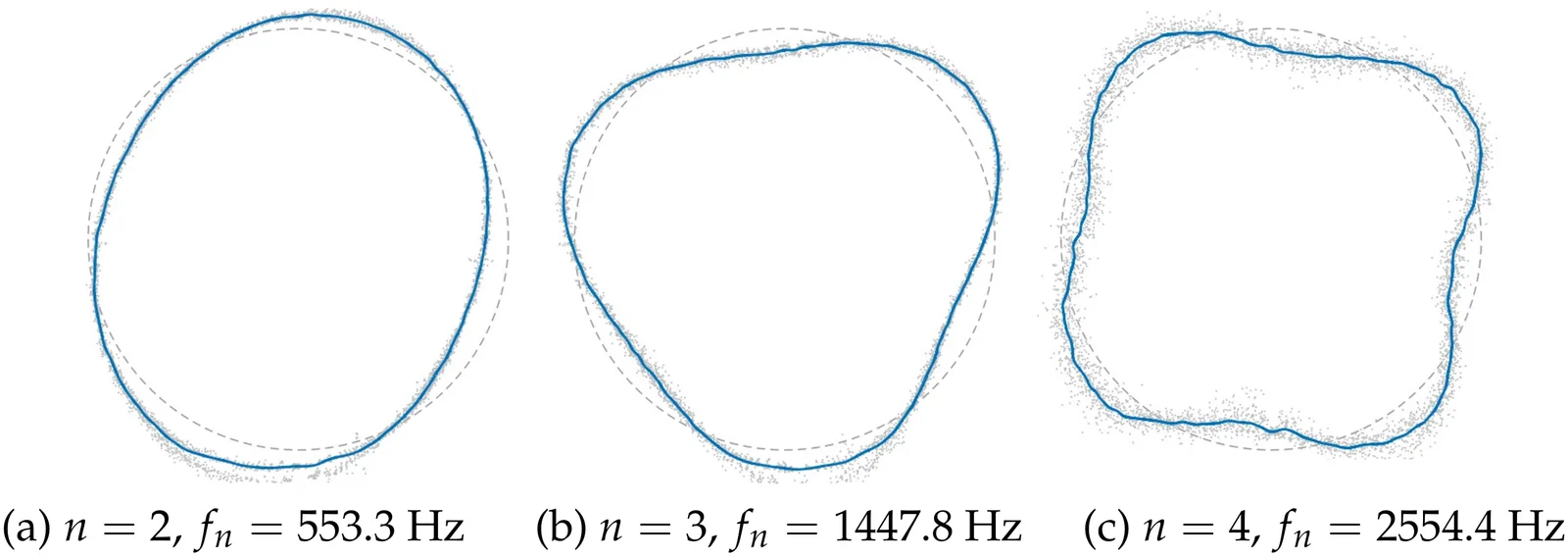
Free-state vision-measured circumferential mode shapes: (**a**) n=2, 553.3 Hz; (**b**) n=3, 1447.8 Hz; (**c**) n=4, 2554.4 Hz. Grey dots, blue curves and grey dashed circles denote phase-projected responses, denoised mode shapes and reference circles, respectively.

Following this OPWD processing, Figure 4 shows the end-face circumferential shapes at 553.3, 1447.8 and 2554.4 Hz (grey dots, discrete pixel responses after phase projection; blue curves, wavelet-denoised shapes). Their circumferential wavenumbers are 2, 3 and 4, giving the orders n=2,3,4; target-mode matching is completed with the LDV candidate frequencies.

### 2.3. Laser Doppler Vibrometry and Frequency Verification

**Figure 5:**
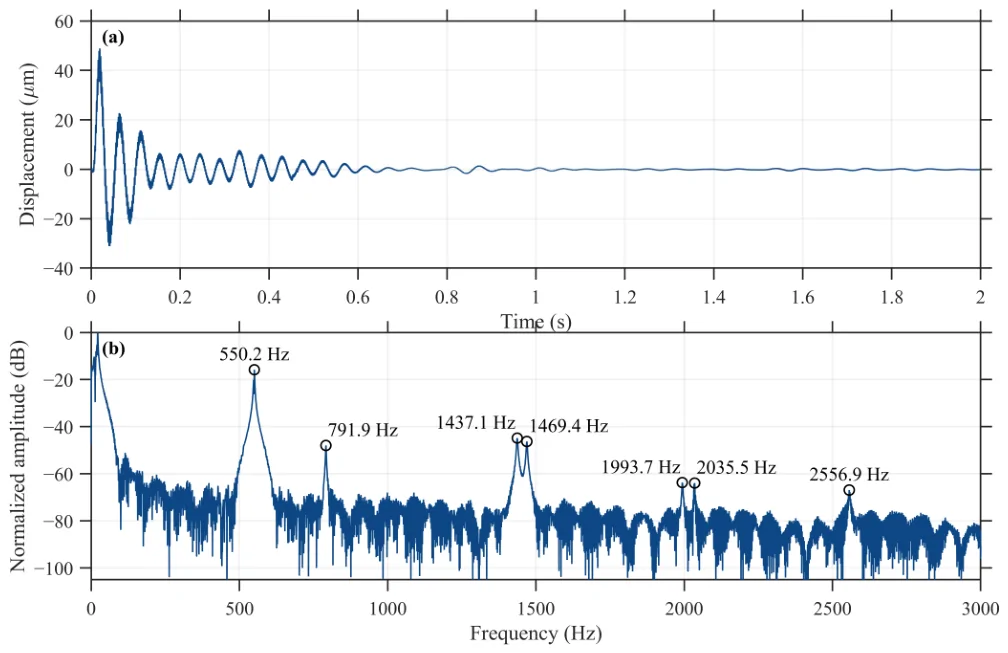
Laser Doppler vibrometry (LDV) results: (**a**) decaying displacement time history; (**b**) one-sided amplitude spectrum, with the candidate frequency peaks annotated.

A valid segment of the decaying LDV displacement record is extracted, mean-subtracted and windowed to compute the one-sided amplitude spectrum (Figure 5), where the main elastic-mode peaks are annotated over the 550.2–2556.9 Hz band; these include both end-face-dominated shell modes and mixed modes with significant axial bending.

Combined with the vision results, the responses near 550.2, 1437.1 and 2556.9 Hz show end-face circumferential shapes of n=2, 3 and 4 and are identified as the three target shell modes. Circumferential shell modes are more sensitive to the wall-thickness variation caused by material removal than axial beam modes, so they are selected as targets for material-parameter updating to establish a free-state baseline for subsequent machining-dynamics analysis.

The 791.9, 1993.7 and 2035.5 Hz peaks are excluded from updating and reserved as non-target modes for the frequency-prediction validation in Section 4; these three non-target modes are selected for independent predictive checks based on peak clarity and modal separability.

Table 1 compares the vision and LDV frequencies; their cross-method deviations are below 0.8%. Vision mainly determines end-face shapes and circumferential orders, while LDV provides higher-resolution frequency measurements. Differences may reflect analysis windows, spatial weighting, neighbouring responses, uneven illumination and image noise. These factors jointly influence the cross-method identification discrepancies, which are used here to assess consistency between the measurement methods. Repeated-measurement statistics for vision and LDV are summarised in Table 2.

With the specimen support and measurement arrangement kept unchanged, three independent impacts were applied and the responses were acquired separately. The specimen remained in the same installed position throughout the three tests. Table 2 reports the mean of the three results and its 90% confidence interval to characterise repeated-impact measurement repeatability within a single setup.

Processing was identical to that used for Table 1. Given the small sample size and unknown population standard deviation, the 90% confidence interval for the population mean was calculated based on Student’s *t*-distribution with df=2, using the two-sided critical value $t_{0.05,2}=2.920$. The interval is $\bar{x}\pm t_{0.05,2}s/\sqrt{N}$, where $\bar{x}$ and *s* are the sample mean and sample standard deviation, respectively, and N=3 is the number of repetitions. The relative deviations between the vision and LDV mean frequencies are 0.16%, 0.48% and −0.16% for the three target modes, all below 0.5% in absolute value; the central frequencies listed in Table 1 fall within the corresponding 90% confidence intervals.

## 3. Finite-Element Modelling of the Thin-Walled Cylinder

### 3.1. Geometric Reconstruction and Deviation Assessment

Geometric reconstruction affects modal prediction. A Creality CR-Scan Raptor Pro hand-held laser scanner measures the specimen surface, saved as a watertight triangular-facet STL model. Manufacturer specifications state an accuracy of up to 0.02 mm and volumetric accuracy of 0.02 mm + 0.08 mm/m in blue-laser mode (https://www.creality.com/support/creality-raptor-pro, accessed on 9 September 2026). This nominal volumetric accuracy is used to assess the Type B standard uncertainty component associated with the scanner, while the radial CAD-STL reconstruction deviation assesses reconstruction consistency. The two assessments cover the device-specification component and the CAD reconstruction stage, respectively, forming the assessed portions of the overall metrological error budget. At a measurement scale of 140 mm, the corresponding specification limit is a=0.02+0.08×0.140=0.0312 mm. Taking ±a provisionally as an estimate of the device error interval and assuming a rectangular distribution within it gives a Type B standard uncertainty component of $u_{B}=a/\sqrt{3}=0.0180$ mm. This estimate is based on the manufacturer’s specifications and the stated distribution assumption. The scan is reconstructed into an editable CAD solid to support FE meshing while retaining measured features within the sampling scale. The steps are as follows.

1. The STL mesh is read, the cylinder axis is identified from the minimum-eigenvalue direction of the area-weighted second moment of facet normals, and the model is rotated onto the global *Z*-axis by a coordinate transformation.
2. Cross-sections are extracted at Δz=2.0 mm axial intervals. Inner and outer contours are identified separately and resampled at common circumferential angles, with $N_{c}=96$ sampling points per contour and angular spacing $\Delta \theta =360^{\circ }/N_{c}=3.75^{\circ }$. Separate contours retain circumferential thickness variation, eccentricity, slight ovality and axial thickness variation within the sampling scale.
3. The outer and inner contours at all axial positions are lofted separately, connecting adjacent sections in axial order into a continuous outer solid and a continuous inner-cavity solid.
4. The inner-cavity solid is subtracted from the outer solid by a Boolean difference, yielding a closed solid with the actual spatial wall-thickness distribution, which is exported in STEP format for the subsequent finite-element modelling.

**Figure 6:**
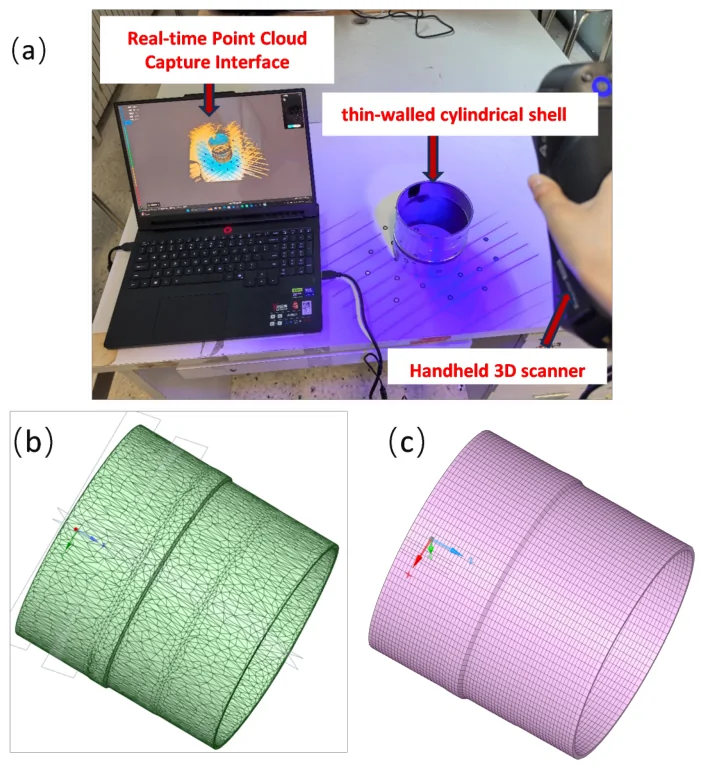
Three-dimensional scanning, geometric reconstruction and mesh discretisation of the thin-walled cylinder. (**a**) Scanning setup; (**b**) triangular facet model; (**c**) finite-element mesh.

The section spacing and circumferential interpolation determine the resolvable scale of local geometric detail. These geometric sampling settings are distinct from the FE mesh size. The scanning setup, triangular-facet model and finite-element mesh are shown in Figure 6.

The scanned STL provides the geometric reference for assessing how closely the reconstructed STEP reproduces the scan. After alignment, inner- and outer-wall radial contours are extracted in axial layers. Radial deviation is defined as $\Delta R=R_{\mathrm{CAD}}-R_{\mathrm{STL}}$, in mm. A circle fit estimates each layer radius to reduce sensitivity to uneven point distribution; layers with valid sections are retained. This assesses how consistently the CAD model reproduces the scan. Scan-acquisition errors and CAD-reconstruction deviations are assessed separately; calibration or repeated-scan data can further develop the overall error budget.

**Figure 7:**
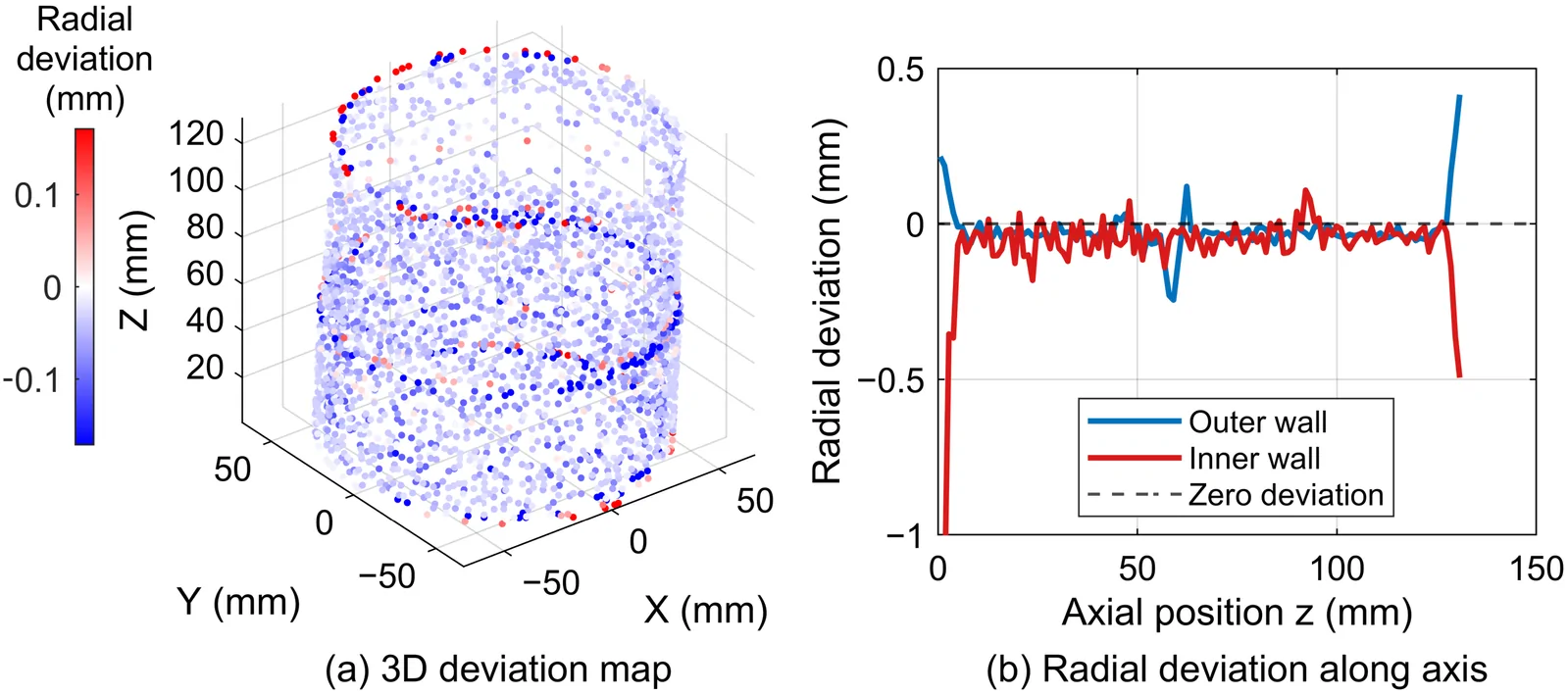
Radial deviation $\Delta R=R_{\mathrm{CAD}}-R_{\mathrm{STL}}$ (mm): (**a**) three-dimensional radial-deviation map; (**b**) axial distributions of the inner- and outer-wall radial deviations.

Figure 7 shows the results: (a) the three-dimensional radial-deviation map and (b) the axial distributions of the inner- and outer-wall deviations. Apart from local rings, deviations over the main wall (z≈5–125 mm) stay within ±0.15 mm, indicating good overall agreement. Larger deviations are concentrated in three regions: the two end edges (end trimming and chamfer differences, reflecting local end features), an outer-wall ring near z≈60 mm (≈−0.4 mm) and an inner-wall ring near z≈88 mm. Over the 238 valid samples within 0–140 mm the median absolute deviation is med|ΔR|=0.0362 mm, about 1% of the mean wall thickness.

### 3.2. Finite-Element Setup and Solution

The reconstructed solid is used to build a linear-elastic, isotropic finite-element model with nominal steel parameters of $E_{0}=210$ GPa, $\rho_{0}=7850$ kg/m^3^ and ν=0.30; $E_{0}$ and $\rho_{0}$ define the nominal baseline and the prior reference for the subsequent regularisation. Geometry and Poisson’s ratio are fixed during updating and only *E* and ρ are adjusted, so the identified parameters effectively absorb part of the residual geometric and boundary errors (see Section 4).

The solid is discretised in ANSYS Mechanical APDL 2026 R1 using ten-node tetrahedral SOLID187 elements. The inner and outer contours and spatial thickness variation are retained. The main solution log records 214,301 nodes and 120,350 elements; the mesh is shown in Figure 6c. To match the sponge-pad flexible support of Section 2, the model uses ideal free–free boundaries to approximate the flexible supports; the contribution of pad stiffness is considered in Section 4.4.

A mesh study uses six nominal sizes from 8 to 2 mm at fixed geometry, free boundaries and material parameters (E=204.93 GPa, ρ=8032.52 kg/m^3^, ν=0.30), with physical modes matched across meshes (Table 3). The effective size is the cube root of the mean element volume. For this homogeneous linear-elastic free-state model at a fixed Poisson’s ratio, changing E/ρ scales all frequencies by the same factor, preserving relative mesh differences. SOLID187 directly retains the reconstructed inner and outer surfaces and spatial thickness variation, simplifying geometry transfer compared with a separate midsurface and thickness-mapping procedure.

Richardson extrapolation with unequal effective-size ratios and a GCI safety factor of 1.25 [35] was applied to the 8, 4 and 2 mm triplet. Under the leading-order asymptotic error model, the 4 mm GCI estimates for n=2,3,4 are 0.083%, 0.120% and 0.161%, respectively, compared with a maximum of 0.361% at 8 mm. A second triplet (2.83, 2.1 and 2 mm) gives a maximum fine-grid GCI of 0.060%; its extrapolated frequencies agree with those from the first triplet within 0.006%. For working-mesh selection, a study-specific tolerance of 0.2% per target frequency allocates a modest discretisation contribution relative to the approximately 1% fitting-error scale. The 4 mm mesh meets this criterion with about 40% of the 2 mm element count and is used for nominal-model calculations and material updating.

**Figure 8:**
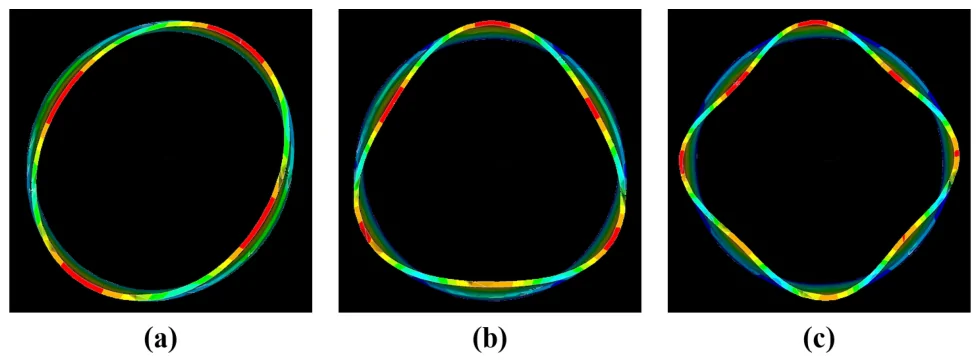
Target mode shapes of the nominal baseline FE model; colours show normalised relative displacement magnitude: (**a**) (1,2) mode; (**b**) (1,3) mode; (**c**) (1,4) mode.

The first 20 modes are extracted with the Block Lanczos method, the first 6 being near-zero-frequency rigid-body modes. Modes are denoted (p,n) by axial and circumferential order, with p=1 as the fundamental (no axial node). Modal correspondence follows three steps: the vision-measured end-face shapes fix the circumferential order *n*, the complete FE mode shapes fix the axial order *p*, and the LDV spectral peaks fix the target frequencies. By the axial nodes and circumferential wavenumbers of the FE mode shapes, the 8th, 12th and 16th modes match the (1,2), (1,3) and (1,4) targets; their end-face views in Figure 8 agree with the vision results.

The nominal baseline frequencies of the (1,2), (1,3) and (1,4) target modes are 555.5, 1477.0 and 2567.7 Hz; the measured reference frequencies are 550.2, 1437.1 and 2556.9 Hz in Table 1 (also used as the optimisation targets; see Section 4.1), so the nominal model overestimates all three (initial errors are tabulated in Section 4). With the geometry, Poisson’s ratio and boundary conditions fixed, Section 4 updates *E* and ρ with regularisation and reports the full before/after frequency comparison.

Geometry and material parameters are treated in stages. Scan-based reconstruction first retains circumferential and axial thickness variations and local geometric features within the sampling scale. CAD-STL radial deviations assess reconstruction consistency, with a median absolute deviation of 0.0362 mm. This geometry is then fixed during material updating. The free–free model approximates the flexible-support experiment by ideal free boundaries, with support stiffness also outside the updating variables. Frequency residuals therefore combine contributions from material scaling, geometric reconstruction, boundary approximation, discretisation and frequency identification. Adjustments to *E* and ρ can compensate for part of these residual discrepancies, yielding effective updated parameters for the adopted geometry and boundary assumptions (Section 4.4).

## 4. Model Updating Based on Non-Contact Experimental Modal Analysis

### 4.1. FE Eigenvalue Problem and E/ρ Degeneracy

The free–free FE model and the updating strategy were established in Section 3: the scan-reconstructed geometry and Poisson’s ratio are fixed, and only Young’s modulus *E* and density ρ are adjusted. Under undamped, linear-elastic small-deformation assumptions, the target modes satisfy the following generalised eigenvalue problem, where K and M are the global stiffness and mass matrices, and $\phi_{i}$ and $f_{i}$ are the mode shape and natural frequency of mode *i*.

(1) $$\left(K-\mu_{i}M\right)\phi_{i}=0,\;\mu_{i}={\left(2\pi f_{i}\right)}^{2}.$$

The dimensionless parameters are $s={\left(s_{E},s_{\rho }\right)}^{T}={\left(E/E_{0},\rho /\rho_{0}\right)}^{T}$, with $E_{0}=210$ GPa and $\rho_{0}=7850$ kg/m^3^. The targets n=2,3,4 correspond to FE modes 8, 12 and 16. The optimisation targets are the recorded LDV peaks of 550.2, 1437.1 and 2556.9 Hz, denoted consistently by $f_{\mathrm{LDV},i}$. These measured peaks are the sole reference for both the optimisation misfit and the relative frequency errors before and after updating. With N=3:

(2) $$J_{\mathrm{mis}}\left(s\right)=\frac{1}{N}\sum_{i=1}^{N}{\left(\frac{f_{A,i}\left(s\right)-f_{\mathrm{LDV},i}}{f_{\mathrm{LDV},i}}\right)}^{2}.$$

This dimensionless form assigns equal weights to the relative frequency residuals; uniform material scaling preserves the spatial mode shapes, which are therefore reserved for a later conformity check. With geometry and Poisson’s ratio fixed, $K=E\hat{K}$ and $M=\rho \hat{M}$ give the scaling relation below, where $g_{i}$ and ${\hat{\phi }}_{i}$ are set by the fixed model. The normalised sensitivities $\partial \ln f_{i}/\partial \ln E=1/2$ and $\partial \ln f_{i}/\partial \ln\rho =-1/2$ (confirmed by a ±1% central-difference check at the nominal point) are collinear, so proportional changes along a fixed-E/ρ ray leave the misfit unchanged: frequencies constrain the ratio E/ρ, while a prior constraint further guides selection of the *E* and ρ combination:

(3) $$f_{i}=\sqrt{E/\rho }\;g_{i},\;\phi_{i}\propto {\hat{\phi }}_{i}.$$

The three target frequencies jointly assess improvement in residuals across circumferential orders through material-scale updating. The collinear sensitivities show that frequencies provide one independent ratio constraint on the two material parameters; the three observations cross-check this scale adjustment across modes. Combining this frequency constraint with the Tikhonov prior selects a stable representative among equivalent parameter combinations, forming a regularised two-parameter update. Geometry and boundaries follow the model established in Section 3; residual geometric and support discrepancies can be partly accommodated by an effective material-scale adjustment. The resulting *E* and ρ are therefore effective parameters for the present model and prior. Independent material information and geometric and boundary observations can further strengthen identification and error separation.

### 4.2. Tikhonov Cost Function and Gradient-Based Solution

A Tikhonov-regularised cost balances frequency fitting against departure from the prior [31,32]:

(4) $$J\left(s;\lambda \right)=J_{\mathrm{mis}}\left(s\right)+\lambda R\left(s\right),\;R={\left(s_{E}-1\right)}^{2}+{\left(s_{\rho }-1\right)}^{2},\;\lambda >0.$$

Here R measures the squared departure of the material scales from nominal and λ controls the prior strength. Since $f_{i}\propto \sqrt{E/\rho }$ makes all combinations along an iso-ratio ray frequency-equivalent (Section 4.1), the penalty varies along that ray and thereby selects a direction among equivalent combinations. Regularisation uses prior information to select a stable solution; the selected *E* and ρ are representative values under the specified prior and weight. The measured specimen mass (≈1.34 kg), together with the reconstructed volume, could in principle provide a density estimate; however, weighing error and the facet-approximation volume deviation are coupled with the geometric reconstruction error, so mass and volume information are used to delimit a reasonable density range.

The bounded problem is solved with the GRAD finite-difference gradients and the FIRST first-order minimisation method of the ANSYS APDL /OPT module [36]. GRAD approximates the partial derivatives directly from objective changes under parameter perturbations; its basic form is

(5) $$\frac{\partial J}{\partial s_{j}}\approx \frac{J\left(s+h_{j}e_{j}\right)-J\left(s\right)}{h_{j}},$$

where $h_{j}$ is the perturbation and $e_{j}$ the *j*-th coordinate unit vector. FIRST builds a search direction from this first-order information and uses a one-dimensional search for a feasible step, updating $s^{k+1}=s^{k}+\alpha_{k}d_{k}$ with direction $d_{k}$ and step $\alpha_{k}$; convergence is judged from changes in the objective and parameters, and one update may require several evaluations.

The weight is chosen by a discrete L-curve: each candidate λ is plotted by its parameter-departure norm $\sqrt{R}$ against the frequency-residual norm $\sqrt{J_{\mathrm{mis}}}$, and a weight in the compromise region of this logarithmic trade-off balances fitting against prior retention. The adopted weight is selected from the fit–prior trade-off across the discrete candidates.

### 4.3. Analysis of the Updating Results

**Figure 9:**
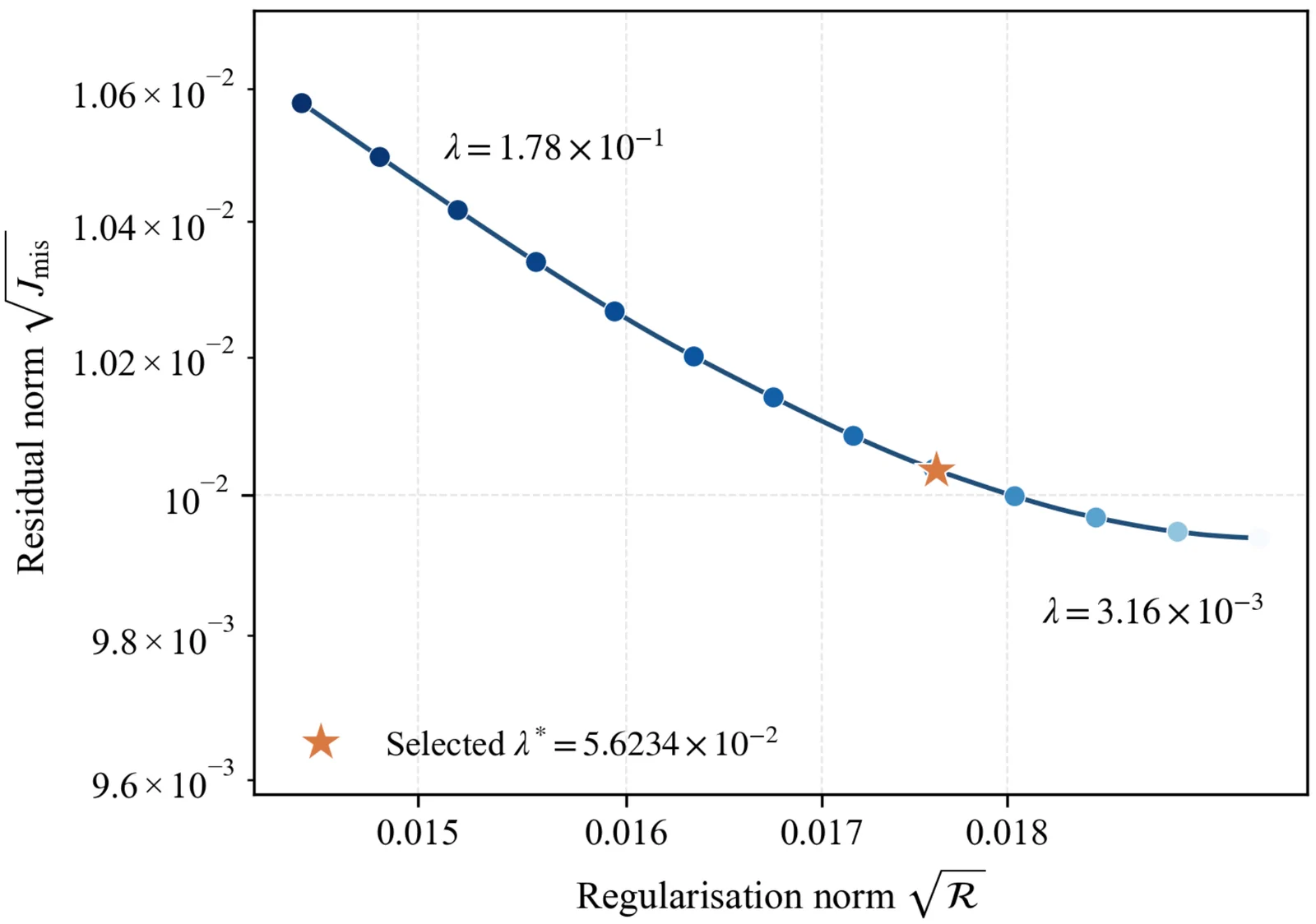
Frequency residual versus parameter departure for discrete regularisation weights. Blue circles represent candidate weights, with darker shades indicating larger values; the blue line connects them, and the orange star marks the adopted weight.

The main /OPT run starts at $\left(s_{E},s_{\rho }\right)=\left(1.04,0.96\right)$, or E=218.4 GPa and ρ=7536 kg/m^3^. These +4% and −4% perturbations place the initial E/ρ above the nominal ratio while remaining within the main bounds $s_{E},s_{\rho }\in \left[0.95,1.05\right]$. These numerical trial values are used to examine the search process. At most 30 outer iterations are used. Figure 9 shows discrete weights over $3.16\times 10^{-3}\leq \lambda \leq 1.78\times 10^{-1}$, with coordinates $\sqrt{R}$ and $\sqrt{J_{\mathrm{mis}}}$. The selected $\lambda^{*}=5.6234\times 10^{-2}$ balances fitting and prior retention.

**Figure 10:**
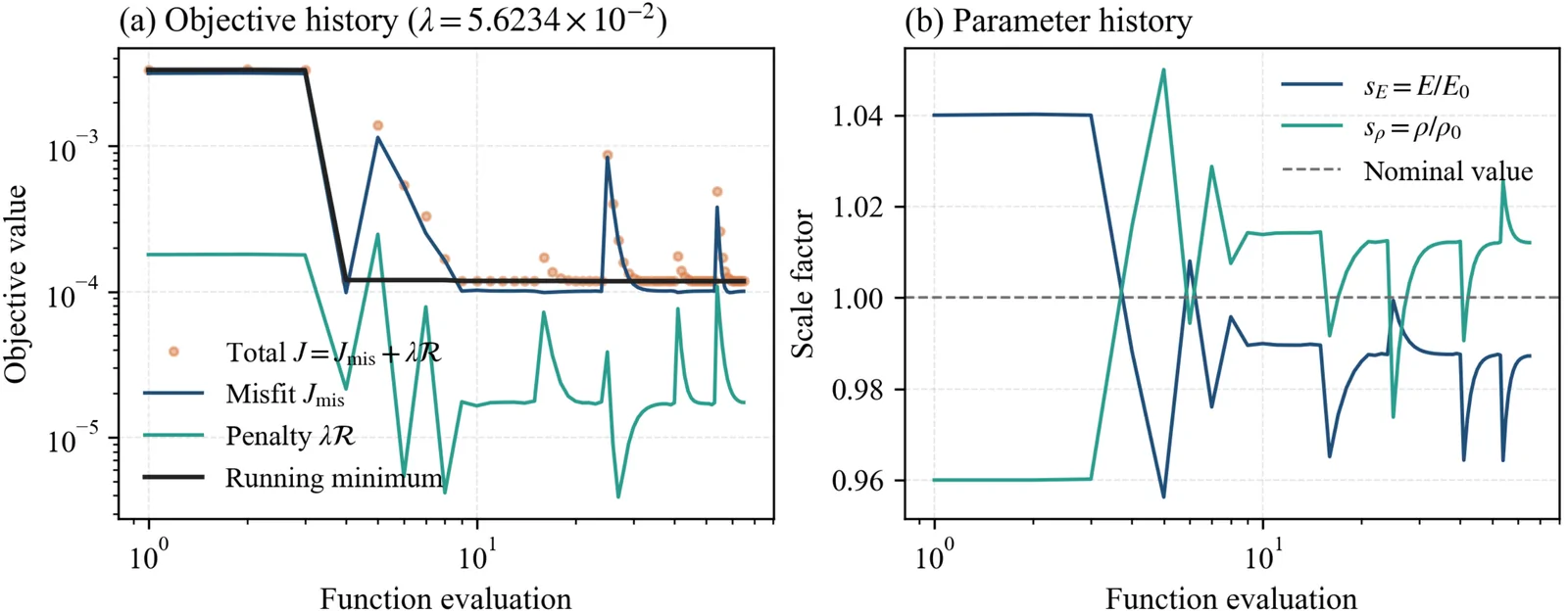
Function-evaluation history of the regularised update: objective components and running minimum (**left**), and material scales (**right**). Horizontal axes show function evaluations on logarithmic scales, including evaluations for gradient perturbations and trial steps.

Figure 10 shows the function-evaluation history at the fixed weight. The left panel tracks the total cost *J*, its misfit $J_{\mathrm{mis}}$ and penalty λR components, and the running minimum of *J*; the right panel tracks the scales $s_{E}$ and $s_{\rho }$ against the nominal value 1. Both use logarithmic evaluation indices, and because the record includes gradient perturbations and trial steps, local spikes are simply higher-cost candidates. As iterations proceed, the perturbations settle into a stationary band and the running minimum stabilises, giving the final scales ≈(0.988,1.012).

**Figure 11:**
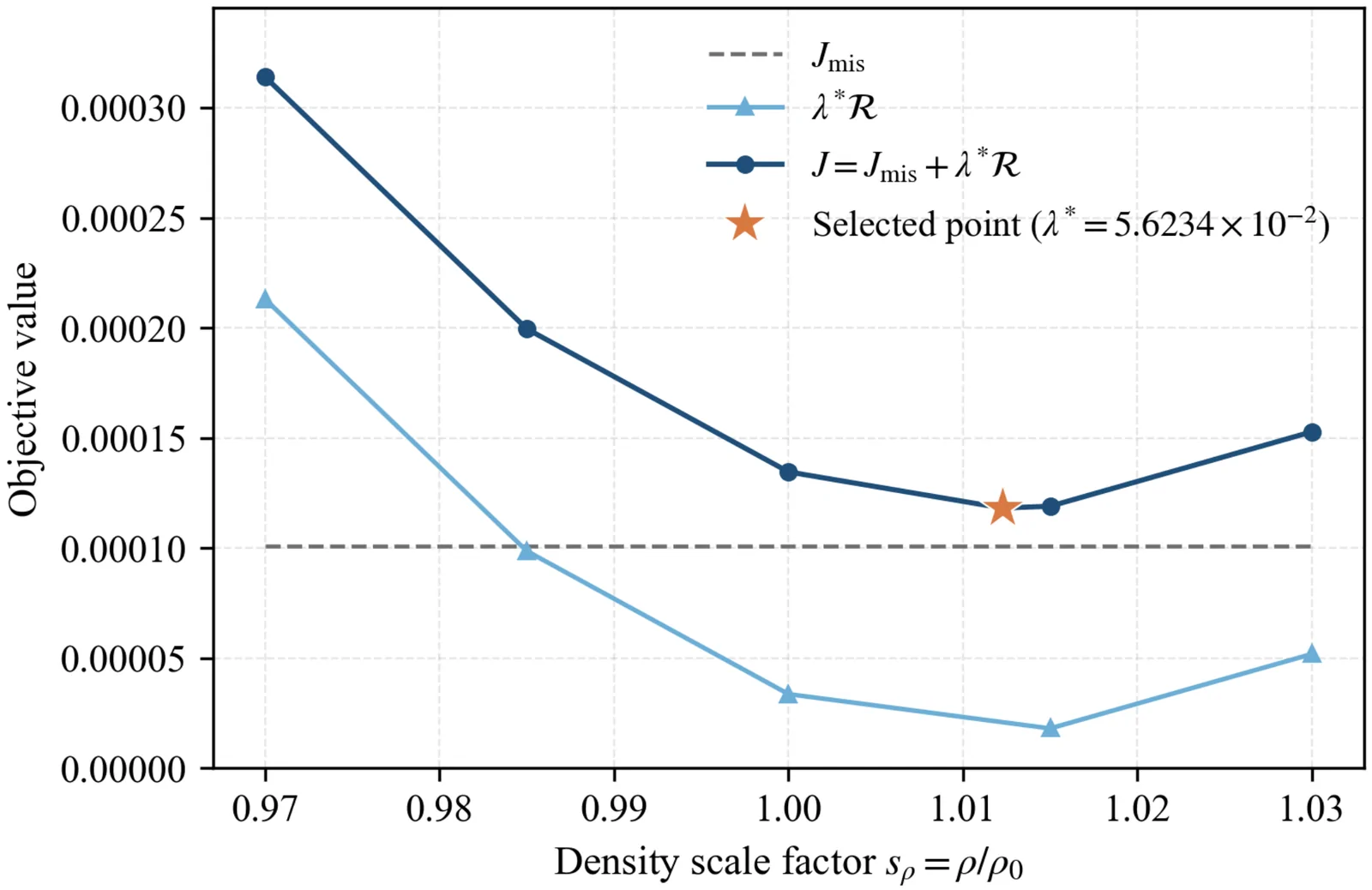
Objective decomposition along an iso-material-ratio ray at fixed regularisation weight.

Figure 11 scans the iso-E/ρ ray at the adopted weight $\lambda^{*}=5.6234\times 10^{-2}$, with abscissa $s_{\rho }=\rho /\rho_{0}$ and *E* varying proportionally so the frequencies stay fixed. Along the ray the misfit $J_{\mathrm{mis}}$ (dashed) is essentially constant (≈1.0 × $10^{-4}$), while the penalty $\lambda^{*}R$ (light blue) decreases monotonically with $s_{\rho }$, so the total cost *J* (dark blue) falls into a flat trough near $s_{\rho }\approx 1.01$–1.02. The star marks the solution selected at the trough’s left edge: where frequency equivalence makes $J_{\mathrm{mis}}$ insensitive to $s_{\rho }$, the prior provides the directional selection, favouring a larger density and the corresponding modulus among the equivalent combinations.

The one-dimensional scan at fixed $\lambda^{*}$ characterises the objective along an iso-ratio ray; a supplementary starting point further examines consistency between search outcomes. A supplementary run starts at $E^{\left(0\right)}=192.0$ GPa and $\rho^{\left(0\right)}=8530$ kg/m^3^, or −8.57% and +8.66% from nominal, placing E/ρ on the lower side. This start is outside the main ±5% bounds. The supplementary bounds are therefore expanded to $s_{E},s_{\rho }\in \left[0.85,1.10\right]$ to examine convergence from the opposite direction over a wider search range. At the same $\lambda^{*}$, the final *E* and ρ differ by about 0.04% and 0.03% (Table 4). The two examined runs give similar endpoints, supporting consistency under the search settings considered.

Table 4 reports parameter endpoints, optimisation statistics and relative RMSE for the two runs. Frequencies are displayed to one decimal place; error metrics and endpoint objectives are calculated from unrounded frequencies and parameters. The final frequencies are (548.6,1458.8,2536.1) Hz and (548.6,1458.7,2535.9) Hz, respectively. Both relative RMSE values are calculated using Equation (6) against the recorded LDV peaks (550.2,1437.1,2556.9) Hz. Here $J_{\mathrm{end}}=J\left(s^{*};\lambda^{*}\right)$ is the dimensionless regularised objective at the endpoint. It combines the frequency misfit against $f_{\mathrm{LDV},i}$ with the parameter-departure penalty and assesses the combined fit–prior trade-off. The running minimum in the convergence plot, $J_{\min}^{\left(m\right)}=\min_{1\leq k\leq m}J\left(s^{\left(k\right)};\lambda^{*}\right)$, is the lowest objective value among the first *m* function evaluations, providing a measure of progress during the search.

Frequency agreement before and after updating is assessed consistently by the root mean square of the relative frequency errors (relative RMSE), using the recorded LDV peaks $f_{\mathrm{LDV},i}$ as reference. For a specified mode group I, define

(6) $$e_{i}=100\frac{f_{A,i}-f_{\mathrm{LDV},i}}{f_{\mathrm{LDV},i}},\;{\mathrm{RMSE}}_{\mathrm{rel}}\left(I\right)=\sqrt{\frac{1}{\left|I\right|}\sum_{i\in I}e_{i}^{2}}\;\left(\%\right).$$

Here $f_{A,i}$ and $f_{\mathrm{LDV},i}$ are the FE frequency and recorded LDV peak for mode *i*, respectively; $e_{i}$ is the signed relative error (%). The set I denotes the evaluated mode group and |I| its number of modes. The target group comprises modes 8, 12 and 16, and the non-target group comprises modes 9, 13 and 14, with |I|=3 in each group.

The lower block of Table 5 contains modes 9, 13 and 14, excluded from fitting. Their relative RMSE decreases from 0.68% to 0.65%. Mode 9 changes from +0.30% to −0.94% with an increase in its error magnitude; reductions in modes 13 and 14 lower the group relative RMSE. The 0.03-percentage-point decrease indicates comparable sub-percent aggregate prediction errors with a small improvement across these three held-out modes. The per-mode results show how the common material scaling redistributes the residuals.

The magnitude of the updating gain depends on the nominal residuals as well as on the parameterisation. On the 4 mm mesh, the target-mode RMSE decreases by 0.71 percentage points (approximately 42% of its nominal value), from 1.71% to 1.00%. Equation (3) shows that material updating adjusts a common frequency scale, whereas mesh refinement changes the mode-dependent baseline frequencies. The resulting fit balances the residuals across modes: the absolute errors decrease for n=2,3 and increase for n=4. The GCI criterion assesses numerical discretisation relative to the limiting FE solution, while the RMSE assesses agreement with experimental frequencies; these complementary measures quantify different contributions to model accuracy.

Finally, measured end-face radial shapes and FE shapes are mapped to a common circumferential grid, normalised and aligned by per-mode circumferential rotation before computing the modal assurance criterion [37]:

(7) $${\mathrm{MAC}}_{ij}=\frac{|\phi_{A,i}^{T}\phi_{\exp,j}{|}^{2}}{\left(\phi_{A,i}^{T}\phi_{A,i}\right)\left(\phi_{\exp,j}^{T}\phi_{\exp,j}\right)}.$$

**Figure 12:**
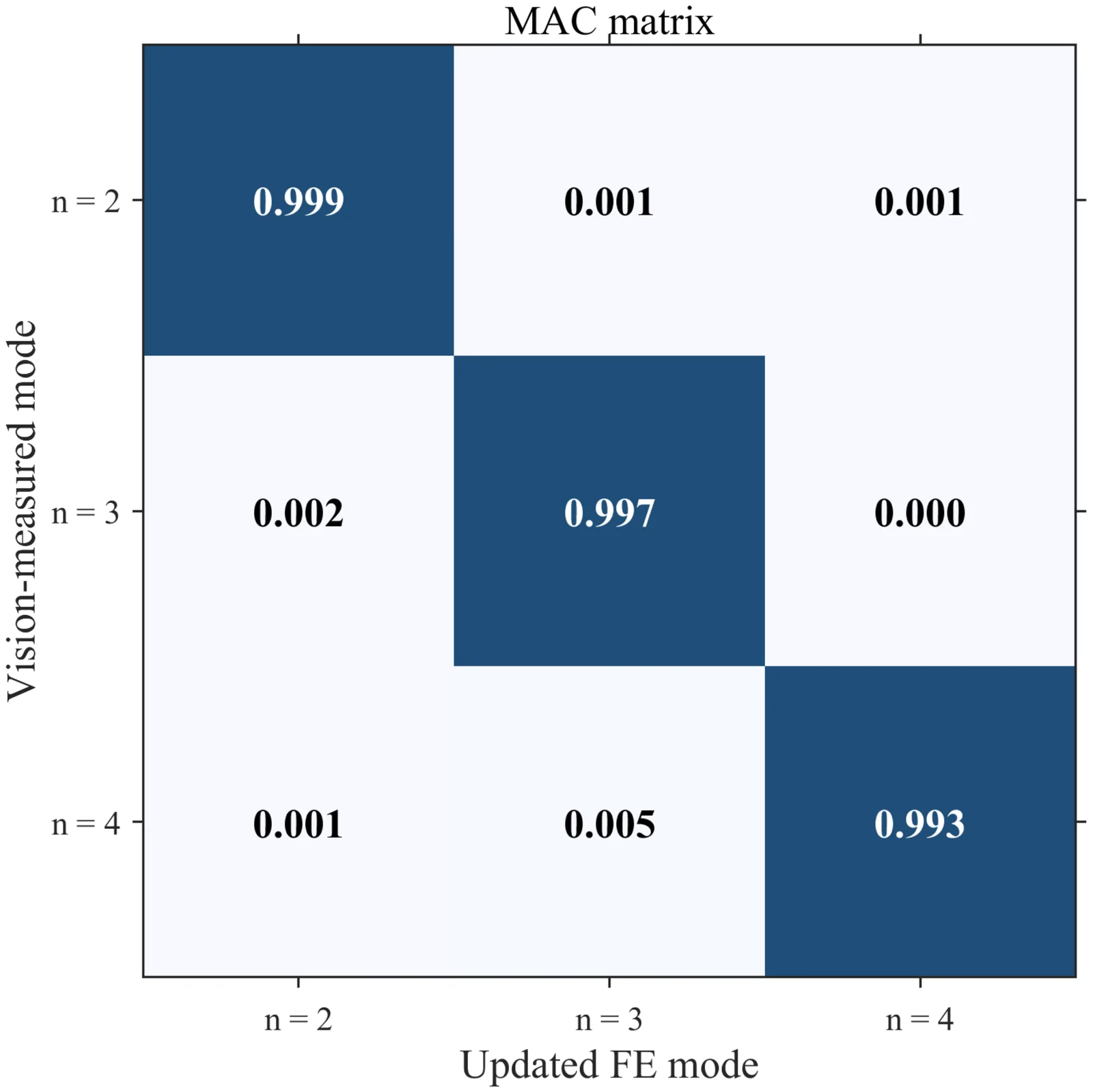
MAC matrix of measured and FE end-face shapes after per-mode circumferential alignment. Darker blue indicates higher MAC values and stronger mode-shape correlation.

In Figure 12, ${\mathrm{MAC}}_{ij}\in \left[0,1\right]$ measures the correlation between FE and measured shapes; the corresponding-mode diagonal values are 0.999, 0.997 and 0.993, with off-diagonal values near zero. Since material scaling preserves mode shapes, MAC provides a complementary check of the morphological agreement of the FE and measured shapes and their consistency with the frequencies.

### 4.4. Error Analysis and Applicability

The reduction in aggregate target-frequency residuals shows an improved overall frequency scale. Independent observations of the contributing stages support attribution of the remaining errors. Possible contributions include scan acquisition, CAD reconstruction, FE discretisation, flexible supports versus ideal free boundaries, and frequency identification. Rated scanner accuracy, CAD-STL deviations, mesh-refinement changes and the frequency confidence intervals from three repeated tests assess different stages and are reported separately. These stage-specific assessments provide a basis for further quantification of error correlations and support-stiffness contributions. With geometry and boundaries fixed, material estimates can compensate for residual errors. Thus $E^{*}$ and $\rho^{*}$ are interpreted as effective updated values for the present model.

The pre-machining baseline is assessed through frequencies and end-face shapes under flexible supports approximating a free state. Scan-based reconstruction supplies the initial geometry, while frequency updating and end-face MAC assess dynamic agreement. Subsequent clamped and post-removal analyses can therefore start from an experimentally assessed model, reducing the influence of initial model discrepancies. In the supplementary calculation of Section 4.5, we have retained the current free-state updated material parameters and applied a deformable-coupled remote displacement constraint on the lateral side to obtain the clamped-state frequencies and mode shapes, illustrating how the free-state baseline model extends to prescribed clamping conditions. Building on this computational demonstration, future work will represent material removal through updated geometry and assess clamped and post-removal predictions against independent measurements, using per-mode relative errors and relative RMSE. Material parameters will remain fixed during validation, focusing the comparison on baseline-parameter transferability and the modelling of boundary and geometric changes. Time-varying cutting conditions will subsequently be incorporated and validated against machining responses. Independent material measurements and support characterisation will further develop the parameter constraints and error budget.

### 4.5. Supplementary Clamped-State Calculation

To examine the application of the free-state updated model under clamped conditions, a supplementary modal calculation is performed on the same thin-walled cylinder, with identical specimen geometry and material state in the free and clamped cases. The free-state updated material parameters E=207.376105 GPa, ρ=7946.444 kg/m^3^ and Poisson’s ratio ν=0.30 are fixed, and only the boundary conditions are modified. A deformable-coupled remote displacement constraint is applied over the rear jaw-contact region: in the working coordinate system, the three translations of the remote point and the rotations about the *X*- and *Y*-axes are set to zero, while the rotation about the *Z*-axis remains free; the front end face remains free. A single unprestressed linear modal solution is performed under the prescribed boundaries. The first 20 modes are extracted with the Block Lanczos method, and FE modes 8, 12 and 16 are matched to n=2,3,4, respectively, from their end-face circumferential shapes.

The free- and clamped-state computed frequencies are compared in Table 6. With the geometry and material parameters unchanged, the remote displacement constraint restricts the overall motion of the clamped region and raises the equivalent stiffness of the corresponding modes, so the frequencies increase. Different modes have different deformation distributions and different sensitivity to the local constraint, so the increases differ among the three modes.

**Figure 13:**
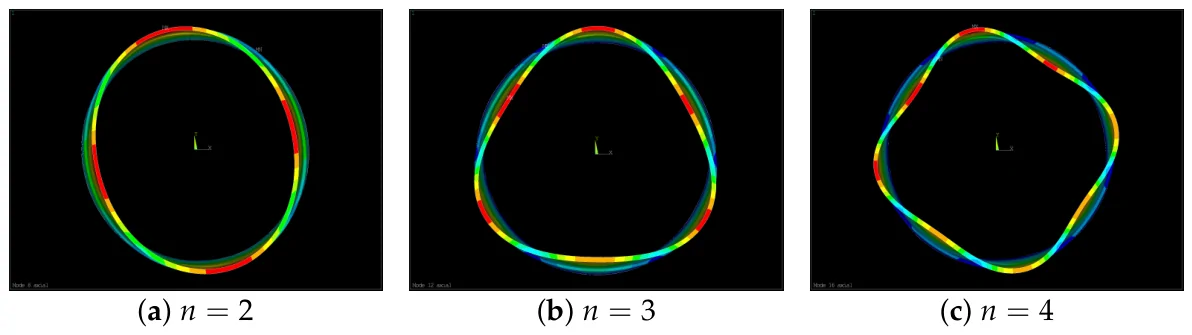
Clamped-state end-face mode shapes under the prescribed remote displacement constraint, corresponding to FE modes 8, 12 and 16, respectively. Colours indicate normalised modal displacement magnitude.

Comparing the free-state shapes in Figure 8 with the clamped-state shapes in Figure 13, the front faces in both states retain the basic elliptical, three-lobed and four-lobed circumferential patterns corresponding to n=2,3,4. The front face remains free in both states, so the selected modes remain dominated by circumferential bending there; the clamping constraint mainly alters the motion of the rear clamped region and the relative deformation distribution between the two end faces. The prescribed remote constraint restricts selected components of the aggregate motion of the rear region while the deformable coupling accommodates local deformation.

This case provides the frequency and mode-shape changes obtained by retaining the free-state updated material parameters and introducing only the clamping boundary, and it offers a computational basis for subsequent identification of the clamping- boundary parameters.

## 5. Conclusions

A free-state baseline modelling workflow of “non-contact measurement—geometric reconstruction—material-property updating” was established for a thin-walled cylinder. Laser vibrometry supplies the target frequencies and vision-based vibrometry the end-face circumferential mode shapes, avoiding the persistent mass loading that contact sensors would impose on the low-stiffness specimen and supporting frequency fitting and mode-shape verification respectively.

Scan-based reconstruction preserves non-axisymmetric thickness within the sampling scale and produces an editable CAD solid and FE model. The median absolute radial CAD-STL deviation is 0.0362 mm over 238 valid samples, about 1% of the mean wall thickness. This measures reconstruction consistency; scanner specifications and CAD-STL deviations characterise scan acquisition and geometric reconstruction, respectively. The maximum target-mode GCI estimate for the 4 mm working mesh is 0.161%, satisfying the study-specific 0.2% criterion under the adopted asymptotic error model.

With the geometry, Poisson’s ratio and free boundaries fixed, frequencies identify the ratio E/ρ. Tikhonov regularisation with a discrete L-curve selects a prior-guided representative from frequency-equivalent parameter combinations; the resulting effective parameters are used to update the present model. Target-mode relative RMSE decreases from 1.71% to 1.00%, while the examined non-target modes retain comparable sub-percent aggregate errors, with a small decrease from 0.68% to 0.65%. Two opposite initial directions under their respective search settings give similar endpoints. End-face MAC values of 0.999, 0.997 and 0.993 support correspondence between the FE and measured target-mode shapes.

Retaining the free-state updated material parameters and applying a deformable-coupled remote displacement constraint on the lateral side, the three computed frequencies are 561.7, 1527.1 and 2659.1 Hz, which are 2.39%, 4.68% and 4.85% higher than the free-state values; the front face still retains the corresponding basic circumferential patterns, while the overall motion of the clamped region is restricted. This numerical case illustrates how the free-state updated model extends to prescribed clamping conditions and defines a basis for subsequent experimental assessment under actual clamping and machining conditions.

This study establishes the pre-machining free-state baseline and assesses its frequencies and end-face shapes, providing an experimentally evaluated starting model for introducing clamping boundaries and post-removal geometry. Future work will refine the description of the clamping boundary and carry out predictions and measured comparisons for the clamped state and after material removal, to assess baseline-model transferability through per-mode relative errors and relative RMSE. Validation will then extend to actual cutting by incorporating time-varying cutting conditions and measured machining responses. Independent material information and assessments of scan and support errors will support this extension.

## Figures and Tables

**Table 1:** Comparison of target-modal frequencies identified by vision vibrometry and laser vibrometry.

Order	Vision (Hz)	LDV (Hz)	Deviation (Hz)	Rel. dev.
n=2	553.3	550.2	+3.1	+0.56%
n=3	1447.8	1437.1	+10.7	+0.74%
n=4	2554.4	2556.9	−2.5	−0.10%

**Table 2:** Target-frequency statistics from three repeated tests (mean ± half-width of the 90% confidence interval).

Order	Vision (Hz)	LDV (Hz)	Deviation (Hz)	Rel. dev.
n=2	550.5±5.6	549.7±1.6	+0.85	+0.16%
n=3	1443.5±6.3	1436.6±1.1	+6.9	+0.48%
n=4	2553.8±3.3	2557.9±3.1	−4.1	−0.16%

**Table 3:** Mesh-refinement results at fixed geometry and material parameters. Nominal sizes are meshing inputs; effective sizes describe the generated meshes.

NominalSize (mm)	EffectiveSize (mm)	Nodes	Elements	n=2 (Hz)	n=3 (Hz)	n=4 (Hz)
8	1.421	107,387	60,003	542.865	1443.882	2511.515
5.66	1.420	107,467	60,063	542.863	1443.880	2511.513
4	1.126	214,301	120,350	542.447	1442.373	2507.496
2.83	1.080	235,595	136,730	542.363	1442.101	2506.956
2.1	0.938	344,162	208,600	542.270	1441.727	2505.958
2	0.828	503,439	302,562	542.217	1441.511	2505.378

**Table 4:** Comparison of the inversion results from two opposite initial directions at the fixed regularisation weight $\lambda^{*}$.

$E^{\left(0\right)}$	$\rho^{\left(0\right)}$	$E^{*}$	$\rho^{*}$	$10^{4}J_{\mathrm{end}}$	${\mathrm{RMSE}}_{\mathrm{rel}}$	FunctionCalls
(GPa)	(kg/m^3^)	(GPa)	(kg/m^3^)		(%)	
218.4	7536	207.376	7946.444	1.18165	1.004	65
192.0	8530	207.300	7944.335	1.18168	1.004	108

**Table 5:** Frequencies, signed relative errors and group relative RMSE before and after updating; the lower block lists the non-target modes excluded from the fit, used for predictive validation.

Mode (FE Order)	LDV (Hz)	Nominal FE (Hz)	Nominal Error (%)	Updated FE (Hz)	Updated Error (%)
Target modes (participating in the fit)					
8 (1,2)	550.2	555.5	+0.96%	548.6	−0.29%
12 (1,3)	1437.1	1477.0	+2.78%	1458.8	+1.51%
16 (1,4)	2556.9	2567.7	+0.42%	2536.1	−0.82%
${\mathrm{RMSE}}_{\mathrm{rel}}$	—	—	1.71%	—	1.00%
Non-target modes (excluded from the fit, for predictive validation)					
9	791.9	794.3	+0.30%	784.5	−0.94%
13	1993.7	2009.1	+0.77%	1984.3	−0.47%
14	2035.5	2052.8	+0.85%	2027.5	−0.39%
${\mathrm{RMSE}}_{\mathrm{rel}}$	—	—	0.68%	—	0.65%

Note: Dashes indicate not applicable.

**Table 6:** Comparison of the three computed free- and clamped-state frequencies.

Circumferential Order *n*	Free-State Frequency (Hz)	Clamped-State Frequency (Hz)	Increase
2	548.6	561.7	2.39%
3	1458.8	1527.1	4.68%
4	2536.1	2659.1	4.85%

## Data Availability

The data presented in this study are available on reasonable request from the corresponding author.
